# Modeling aging and retinal degeneration with mitochondrial DNA mutation burden

**DOI:** 10.1111/acel.14282

**Published:** 2024-08-29

**Authors:** John Sturgis, Rupesh Singh, Quinn R. Caron, Ivy S. Samuels, Thomas Micheal Shiju, Aditi Mukkara, Paul Freedman, Vera L. Bonilha

**Affiliations:** ^1^ Department of Ophthalmic Research, Cole Eye Institute Cleveland Clinic Cleveland Ohio USA; ^2^ Department of Molecular Medicine, Cleveland Clinic Lerner College of Medicine, School of Medicine Case Western Reserve University Cleveland Ohio USA; ^3^ Research Service Louis Stokes Cleveland VA Medical Center Cleveland Ohio USA; ^4^ College of Arts and Sciences Case Western Reserve University Cleveland Ohio USA; ^5^ Department of Ophthalmology, Cleveland Clinic Lerner College of Medicine, School of Medicine Case Western Reserve University Cleveland Ohio USA; ^6^ Present address: Debusk College of Osteopathic Medicine Knoxville Tennessee USA

**Keywords:** D257A, mitochondria, mitochondrial DNA (mtDNA), polymerase gamma (POLG), retina, retinal degeneration

## Abstract

Somatic mitochondrial DNA (mtDNA) mutation accumulation has been observed in individuals with retinal degenerative disorders. To study the effects of aging and mtDNA mutation accumulation in the retina, a polymerase gamma (POLG) exonuclease‐deficient model, the Polg^D257A^ mutator mice (D257A), was used. POLG is an enzyme responsible for regulating mtDNA replication and repair. Retinas of young and older mice with this mutation were analyzed in vivo and ex vivo to provide new insights into the contribution of age‐related mitochondrial (mt) dysfunction due to mtDNA damage. Optical coherence tomography (OCT) image analysis revealed a decrease in retinal and photoreceptor thickness starting at 6 months of age in mice with the D257A mutation compared to wild‐type (WT) mice. Electroretinography (ERG) testing showed a significant decrease in all recorded responses at 6 months of age. Sections labeled with markers of different types of retinal cells, including cones, rods, and bipolar cells, exhibited decreased labeling starting at 6 months. However, electron microscopy analysis revealed differences in retinal pigment epithelium (RPE) mt morphology beginning at 3 months. Interestingly, there was no increase in oxidative stress and parkin‐mediated mitophagy in the ages analyzed in the retina or RPE of D257A mice. Additionally, D257A RPE exhibited an accelerated rate of autofluorescence cytoplasmic granule formation and accumulation. Mt markers displayed different abundance in protein lysates obtained from retina and RPE samples. These findings suggest that the accumulation of mtDNA mutations leads to impaired mt function and accelerated aging, resulting in retinal degeneration.

## Graphical abstract

This manuscript provides the first retinal characterization of the D257A mouse model. D257A mice display accelerated age‐related morphological and functional deficits in multiple retinal cell types, with specific emphasis on the photoreceptors and retinal pigment epithelium (RPE). The phenotypes presented are promoted by the depletion of essential mitochondrial electron transport proteins in both the retina and RPE.

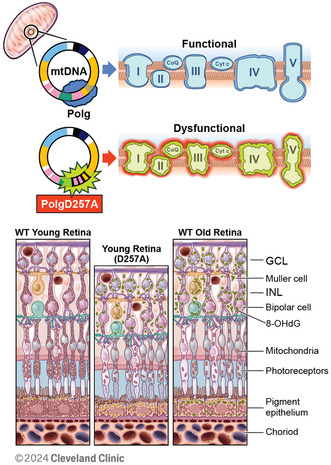

**Abbreviations**
4‐HNE: 4‐hydroxynonenal
8‐OHdG: 8‐hydroxy‐2′‐deoxyguanosine
AF: autofluorescent
AMD: age‐related macular degeneration
C I: complex 1
C II: complex 2
C III: complex 3
C IV: complex 4
C V: complex 5
CIS: cone inner segment
CTCF: corrected total cell fluorescence
D257A: Polg^D257A^ mutator mice
DIC: differential interference contrast
DJ‐1: Parkinson's disease protein 7
DNPH: 2,4‐dinitrophenylhydrazine
ERG: electroretinography
ETC: electron transport chain
GATD3: glutamine amidotransferase class 1 domain containing 3
GCL: Ganglion cell layer
INL: inner nuclear layer
IPL: inner plexiform layer
IS: inner segments
LC3B: microtubule‐associated proteins 1A/1B light chain 3B
MnSOD: manganese‐dependent superoxide dismutase
Mt: mitochondrial
mtDNA: mitochondrial DNA
NNT: nicotinamide nucleotide transhyrdogenase
OCT: optical coherence tomography
ONL: outer nuclear layer
OPL: outer plexiform layer
OS: outer segments
P62: sequestosome 1
PDE6C: phosphodiesterase 6C
PDHA1: pyruvate dehydrogenase
PGAM5: phosphotase phosphoglycerate mutase 5
PGC1a: peroxisome proliferator‐activated receptor gamma coactivator 1‐alpha
PKCa: protein kinase C alpha
POLG: polymerase gamma
RIS: rod inner segment
RNFL: retina nerve fiber layer
RPE: retinal pigment epithelium
rRNA: ribosomal RNA
SD: standard deviation
SD‐OCT: spectral‐domain optical coherence tomography
Ser: serine
SNVs: single‐nucleotide variants
TEM: transmission electron microscopy
Thr: threonine
tRNA: transfer RNA
VDAC: voltage‐dependent anion channel
VIP: volume intensity projection
WT: wild‐type

## 1 INTRODUCTION

Mitochondria are unique eukaryotic organelles that possess their own mt genome containing approximately 16.5 kb of DNA. These genes encode several subunits of the electron transport chain (ETC), including 13 proteins, 22 transfer RNAs (tRNA), and two ribosomal RNAs (rRNA), which play a vital role in oxidative phosphorylation and energy production (Chinnery & Hudson, 2013). Genetic instability of the mitochondrial (mt) genome can occur through various pathways, such as base‐substitution mutations, deletions, failure of base‐excision repair, and oxidative insult (Kujoth et al., 2007). Additionally, mtDNA is more susceptible to damage due to its high replication rate, proximity to free radical production sites, the lack of protective histones, and higher coding density (Kennedy et al, 2013; Nissanka & Moraes, 2018). Recent research into mtDNA mutations has revealed that their frequency increases with age and varies among tissues (Sanchez‐Contreras et al., 2023).

The retina, a post‐mitotic tissue responsible for vision, comprises many cell types, all heavily reliant on the functions of mitochondria for health and survival. Mitochondria have several essential roles that maintain cell health, including energy generation, regulation of calcium levels and cholesterol and iron metabolism (Ferrington et al., 2020). Mt dysfunction in one or more retinal cell types has been implicated in human retinal degenerative diseases such as age‐related macular degeneration (AMD), retinitis pigmentosa, diabetic retinopathy, and glaucoma (Eells, 2019). The retinal pigment epithelium (RPE) is a monolayer of cells located at the outer boundary of the retina and contains abundant mitochondria. The RPE regulates the transport of nutrients from the choroidal blood supply to the neural retina. It relies on reductive carboxylation and oxidative phosphorylation for energy production, while the retina relies on glycolysis (Du et al., 2016; Hurley, 2021). As we age, RPE mitochondria become dysfunctional, upsetting the established balance between the RPE and retina. This imbalance is thought to be accelerated in disease states, with damaged RPE mt driving the onset and progression of retinal degeneration or AMD (Ferrington et al., 2020; Hurley, 2021; Kaarniranta et al., 2020). The accumulation of mtDNA mutations has been identified as a source of mt dysfunction when analyzing normal and diseased human donor tissue. In addition, mtDNA mutation burden in the RPE has been linked to both disease stage and likelihood of progression in AMD (Karunadharma et al., 2010). Cumulatively, these observations have provided a novel mechanism to explore in the context of retinal degenerative diseases.

The current study uses a previously developed mouse model of accelerated aging known as D257A mutator (D257A) mouse to understand the role of mtDNA mutation accumulation in the retina. This model has a single amino acid substitution (D257A) in the exonuclease domain of the polymerase gamma (POLG) protein responsible exclusively for mtDNA replication and repair. This mutation does not impact the replicative capabilities of POLG, but it impairs its ability to perform base‐excision repair, resulting in an approximately 2500‐fold increase in mtDNA mutations (Kujoth et al., 2005). Since its development, this model has been widely used as a tool to study aging and accelerated mtDNA mutation burden across various tissues, including brain, liver, and skeletal muscle (Dai et al., 2014; Hiona et al., 2010; Maclaine et al., 2020). Importantly, a previous study used Duplex sequencing to identify organ‐specific mtDNA mutation burden in heart, skeletal muscle, eye, kidney, liver, and brain in naturally aged mice at 4.5 and 26 months of age (Sanchez‐Contreras et al., 2023). An initial comparison of the mtDNA single‐nucleotide variants (SNVs) frequency in young mice revealed a mutation frequency of ~1 × 10^−6^, with low tissue variability. However, with age, significant increases in SNV frequency in all tissues in the aged cohort were observed, with the kidney having the highest SNV frequency (6.60 ± 0.56 × 10^−6^) and the heart having the lowest (1.74 ± 0.16 × 10^−6^), RPE/choroid was similar to liver and displayed a trend of being slightly higher than the retina. Moreover, the observed changes in frequency with age or tissue type did not correlate with differences in mtDNA copy number, as the mtDNA:nDNA ratio did not change with age.

However, the retina or RPE has never been investigated in the context of this model. Mutations in POLG have commonly been associated with neurodegenerative diseases such as Alpers‐Huttenlocher syndrome, childhood myocerebrohepatopathy, ataxia neuropathy spectrum, and autosomal progressive external ophthalmoplegia (Rahman & Copeland, 2019). Other diseases associated with specific mtDNA point mutations include but are not limited to Leber's hereditary optic neuropathy, mitochondrial encephalopathy, and myoclonic epilepsy (Zeviani & Carelli, 2022). This study aims to elucidate the role of D257A‐mediated mtDNA mutation accumulation in retinal aging. It uses diverse and complementary methodologies to test the hypothesis that the accumulation of mtDNA mutations plays a causative role on the onset and progression of retinal degeneration. As the first study to employ the D257A mutator mouse with consideration for the retina, this analysis characterizes the effects of mtDNA mutations on retinal mt structure, function, and health.

## 2 RESULTS

### 2.1 Accelerated age‐related morphological and functional retinal deficits in the D257A mice

**FIGURE 1:**
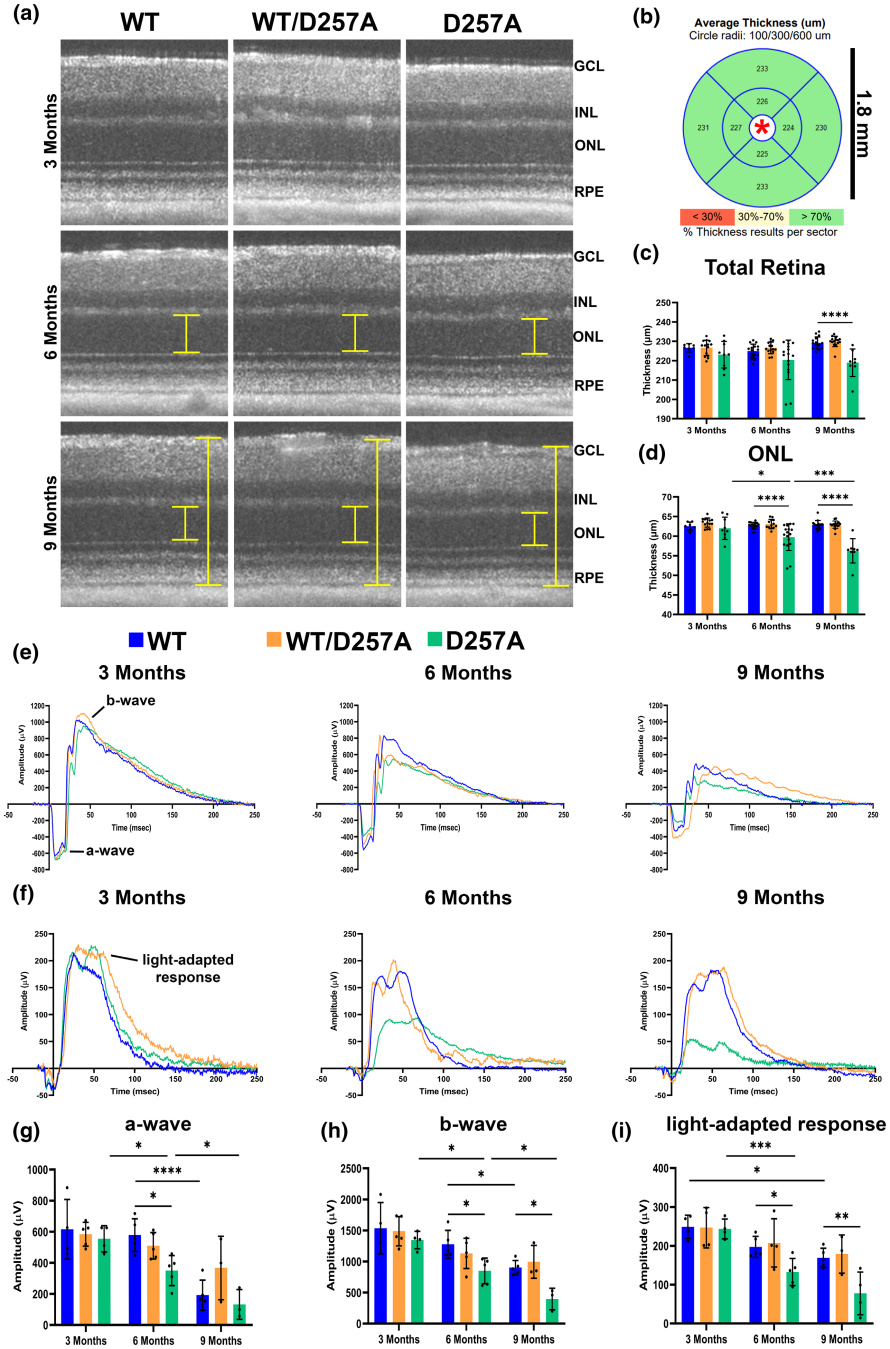
In vivo morphological and functional retinal characterization of D257A retinas. (a) Averaged representative raw OCT image files (yellow bars = layers of the retina where thickness differences were observed). (b) Representative heat map assembled by InVivoVue software used for downstream analysis (only green regions representing >70% thickness results per sector were quantified for accuracy); *is centered on the optic nerve head; area analyzed = 1.8 mm × 1.8 mm. (c) Graphical representation of total retinal thickness and (d) outer nuclear layer thickness (ONL). Representative wave‐form traces of dark‐ (e) and light‐adapted (f) electroretinography (ERG) in response to flash stimulus (1.4 log cd·s/m^2^). Graphs of the amplitude of the a‐wave (g), b‐wave (h), and light‐adapted response (i) to the flash luminance at 24.1 cd·s/m^2^. Data are expressed as mean ± SD. *p ≤ 0.05, **p ≤ 0.01, ***p ≤ 0.001, ****p ≤ 0.0001; two‐way ANOVA. Data points represent biological replicates; asterisks indicate significance, *n* = 3–5.

To investigate the role of mtDNA mutation burden on the retina, optical coherence tomography (OCT) was performed using a previously established protocol (Singh et al., 2020). Imaging was performed on WT (wild‐type, WT), WT/D257A (heterozygous), and D257A/D257A (D257A) littermate mice at 3‐, 6‐, and 9‐months of age (Figure 1a). Raw images were then averaged, central and peripheral to the optic nerve, and heat maps were generated and quantified (Figure 1b). Over the observed time course, the total retinal thickness of WT and WT/D257A mice did not significantly change. However, the total retinal thickness of the D257A mice was significantly decreased (~10.27 μm) (Figure 1c). Individual layers were also quantified and the outer nuclear layer (ONL), which contains the nuclei of the cone and rod photoreceptors, showed a significant decrease in thickness by 6 months of age and continued to be significantly decreased at 9 months of age (2.96 μm and 6.63 μm, respectively). (Figure 1d). Other retinal layers showing differences at specific ages included the RPE, photoreceptor outer segments (OS), inner nuclear layer (INL) and retina nerve fiber layer (RNFL) (Figure S1A). Electroretinography (ERG) was performed to study the effect of mtDNA mutation burden on retinal cells' response to light flashes using a previously established protocol (Aiello et al., 2023). At 6 months of age, D257A mice exhibited a significant decrease in the amplitude of the a‐wave (Figure 1e,g), indicating a functional decline in rod photoreceptor cells. This response was also significantly reduced when comparing D257A mice at ages 3, 6, and 9 months, whereas WT mice only displayed a significant decrease between 6 and 9 months. Furthermore, D257A mice demonstrated reduced b‐wave amplitude at 6 and 9 months old (Figure 1e,h); however, this difference seemed to be dependent on the reduced a‐wave amplitude and not because of loss of bipolar cell function themselves (Figure S1B). Finally, the D257A mice showed a significant decrease in their light‐adapted response, indicating a reduction in cone photoreceptor cell health. This response was significantly diminished in D257A between 3 and 6 months but in WT only between 3 and 9 months (Figure 1f,i). This result was significant, as cone photoreceptors were shown to rely significantly more on mt function than their rod counterparts in previous literature (Li et al., 2020). These results indicate that D257A mice experience retinal thinning in the photoreceptor layer at an earlier age than WT. Additionally, all recorded responses identify a functional compromise in the D257A mice that appears earlier than WT, with the most severe phenotype observed in the cone photoreceptor's function. Since there were little to no observed significant differences between the WT and WT/D257A compared to D257A mice using both modalities, moving forward, D257A mice were only compared to WT.

### 2.2 Accelerated age‐related changes in the retina of the D257A mice

**FIGURE 2:**
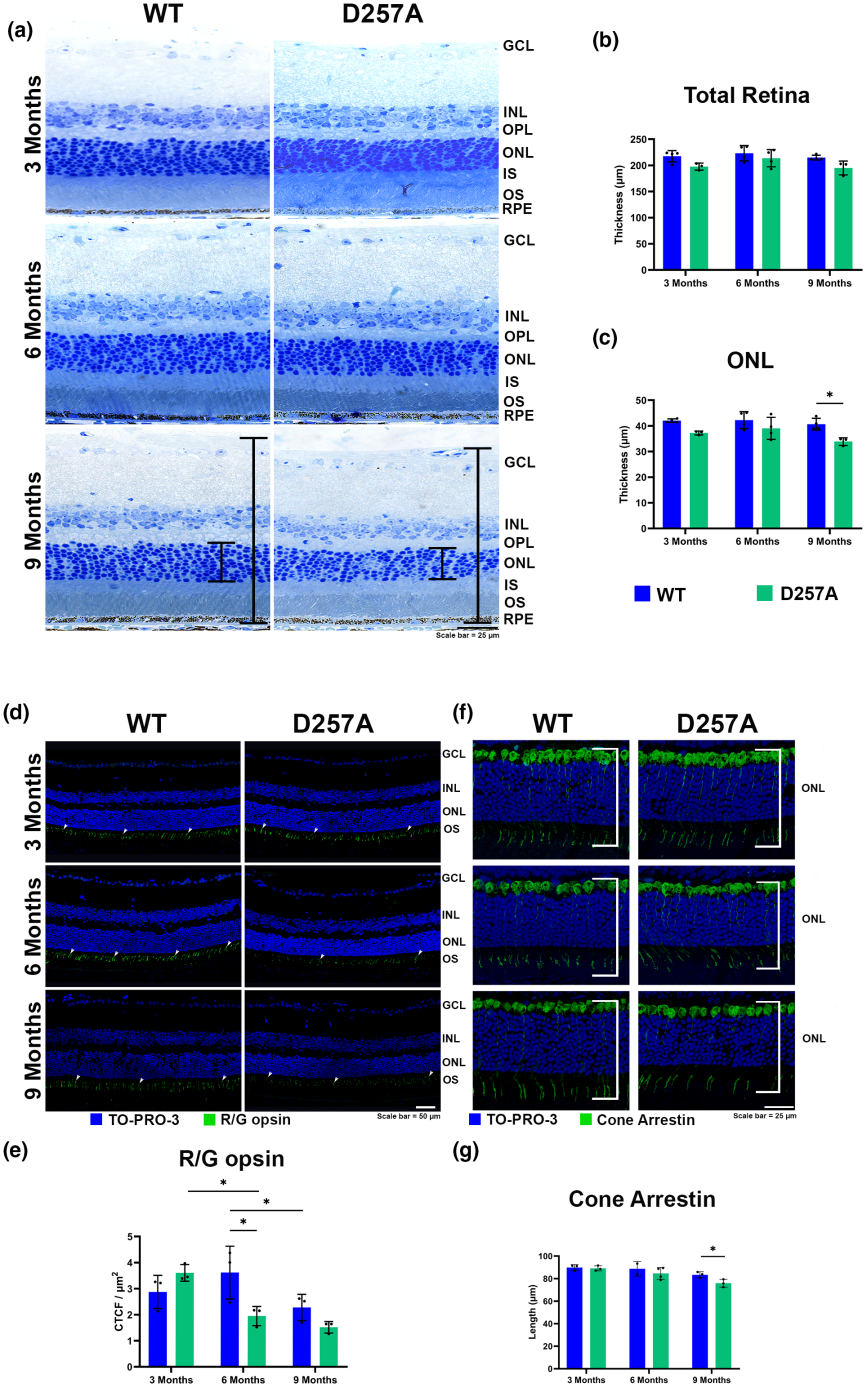
Loss of normal retinal morphology and essential proteins in the D257A mouse. (a) Representative retinal histological sections stained with toluidine blue. (b) Graphical representation of measured total retinal thickness and (c) outer nuclear layer (ONL) thickness. (d) Immunofluorescence staining of cone photoreceptor outer segment marker R/G opsin. (e) Graphical representation of R/G opsin staining. (f) Immunofluorescence staining of cone arrestin marker. (g) Graphical representation of cone arrestin staining. Data are expressed as mean ± SD. *p ≤ 0.05; two‐way ANOVA, student's *t*‐test. Data points represent biological replicates; asterisks above for significance.

Histology and immunohistochemistry with retinal cell markers were performed to further investigate the in vivo morphological and functional data observed in the D257A retina. Retinal histological sections revealed a trend of decline in total retinal thickness, which was particularly significant in the ONL of the photoreceptors by 9 months of age (Figure 2a, brackets and 2b,c). Furthermore, D257A mice retinas probed with an R/G opsin antibody, a commonly used marker for cone photoreceptor outer segments (Molday et al., 2019), showed reduced labeling for this protein by 6 months when compared to WT. This marker was significantly decreased between 3‐ and 6‐month‐old D257A, whereas a significant reduction in WT was detected between 6 and 9 months of age (Figure 2d, arrowheads, 2e). Retinas were also probed with a cone arrestin antibody, a pan cone marker that labels cones from the synaptic terminals to the outer segments (Figure 2f). Measurements of the cone thickness using this labeling detected a significant reduction in cone length at 9 months in the D257A (Figure 2g). Furthermore, utilizing rhodopsin and protein kinase C alpha (PKCα) antibodies, commonly used markers for rod photoreceptor OS and bipolar cell synapses, respectively (Molday et al., 2019), these labelings displayed a reduced trend at 6 and 9 months of age in D257A retinas (Figure S2A–C). However, a significant reduction could be observed when comparing 3‐ and 9‐month‐old D257A (Figure S2A,B). To assess the effect of D257A on photoreceptor mt, transmission electron microscopy (TEM) analysis was performed on WT and D257A mice at 3 months of age. The D257A cone inner segment (CIS) and rod inner segment (RIS) mitochondria appear more disorganized, less electron‐dense, and improperly aligned with the cell body compared to WT (Figure S2C). Finally, D257A protein lysate showed a non‐significant decrease in the is cone‐specific phosphodiesterase 6C (PDE6C) with age (Figure S2D) in both mice. This data validates our in vivo results, again indicating a more severe phenotype in the cone photoreceptors.

### 2.3 Accelerated age‐related changes in RPE of the D257A mice

**FIGURE 3:**
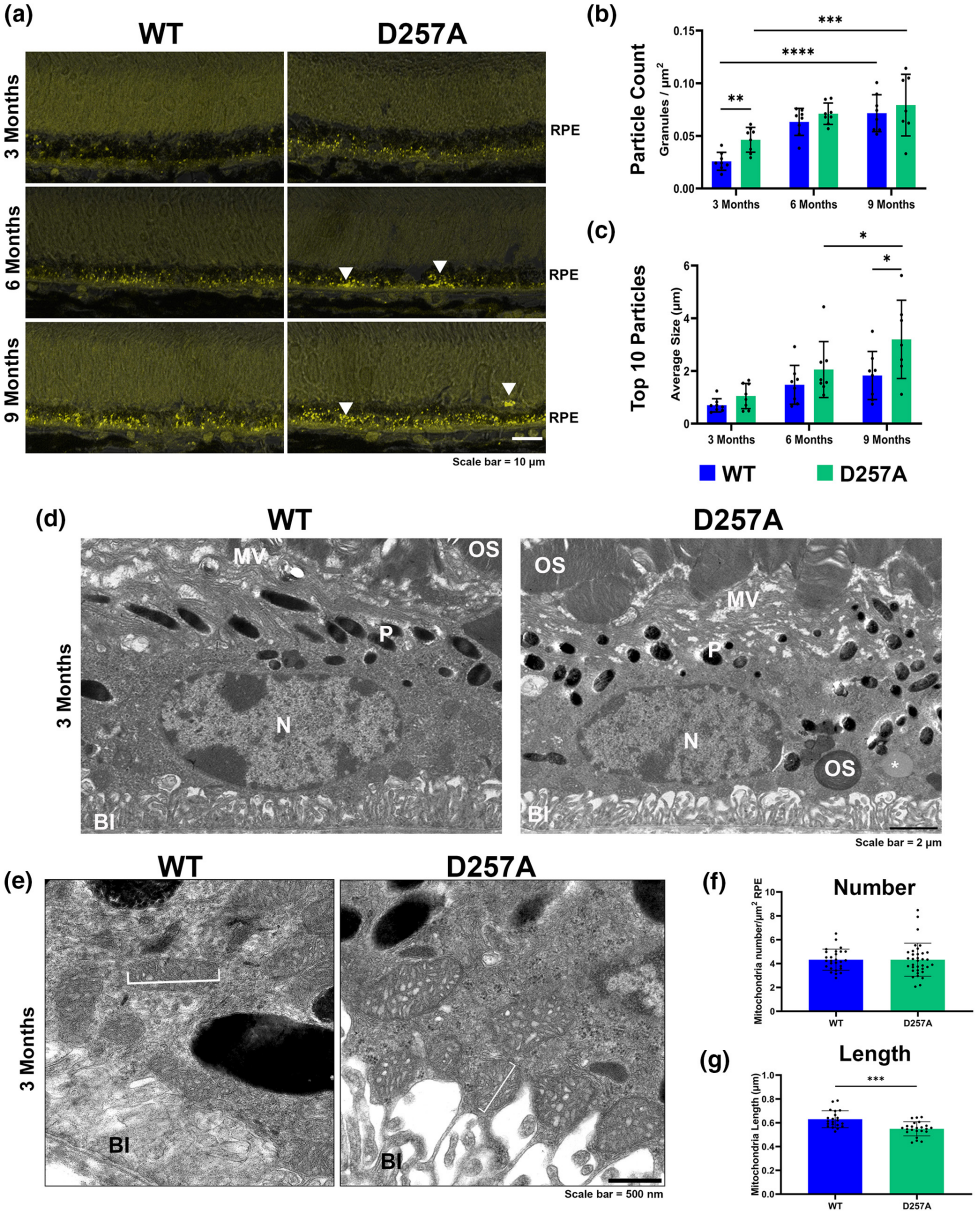
D257A RPE exhibits altered autofluorescent (AF) granule accumulation and size distribution with age. (a) Representative retinal sections excited between 488 and 594 nm wavelength with overlayed bright field to show RPE‐specific AF granule accumulation. White arrowheads represent larger granules. (b) Graphical representation of the number of particles counted in the RPE layer after background thresholding divided by the area of RPE analyzed. (c) Graphical representation of the averaged top 10 particle sizes per image acquired. (d) Representative electron micrographs of 3‐month WT and D257A RPE (*N* = 3). MV, microvilli, P, pigment granule, N, nucleus, OS, outer segment, BI = basal infoldings. (e) High magnification representative electron micrographs of 3‐month WT and D257A mt. (f) Graphical representation of mt number and (g) mt length. Data are expressed as mean ± SD. *p ≤ 0.05, **p ≤ 0.01, ***p ≤ 0.001, ****p ≤ 0.0001; two‐way ANOVA, student's *t*‐test. Data points represent biological replicates and individual mitochondria; asterisks above for significance.

Autofluorescent (AF) granules were observed in the RPE cytoplasm of unlabeled sections. To investigate this observation further, we obtained and analyzed images of the relative amount of AF granules in the RPE cytoplasm. At 3 months, the D257A RPE showed increased AF granule accumulation in their cytoplasm. Additionally, WT and D257A significantly increased the number of AF granules in the RPE when comparing 3‐ and 9‐months of age (Figure 3a, arrowheads, 2b). By 6 months of age, the WT and D257A RPE showed similar levels of AF granule formation (Figure 2b). By 9 months, the D257A RPE had significantly larger AF granules when comparing the top 10 granule sizes per mouse (Figure 3a,c). To better understand the nature of the observed AF granules, TEM analysis was performed at 3 months of age. The D257A RPE showed disorganized microvilli (Figure 3d, MV) and disorganized basal infoldings (Figure 3d, BI). Also, the D257A melanin pigments appeared smaller and disorganized compared to WT's proper apical membrane alignment (Figure 3d, P). Undigested photoreceptor OS near the basal surface were frequently observed in the D257A RPE cytoplasm (Figure 3d, OS). Finally, D257A RPE showed an increased frequency of electron‐lucent vacuoles (Figure 3d, asterisk). At high magnification, the overall amount of mitochondria did not differ between WT and D257A, but the mitochondria in the D257A RPE were significantly shorter than in WT mice (Figure 3e–g). This data suggests that the D257A RPE accumulates more and larger AF granules with age, and changes in overall RPE morphology and mitochondria can be seen as early as 3 months.

### 2.4 Retinal and RPE mt dysfunction in the D257A mice

**FIGURE 4:**
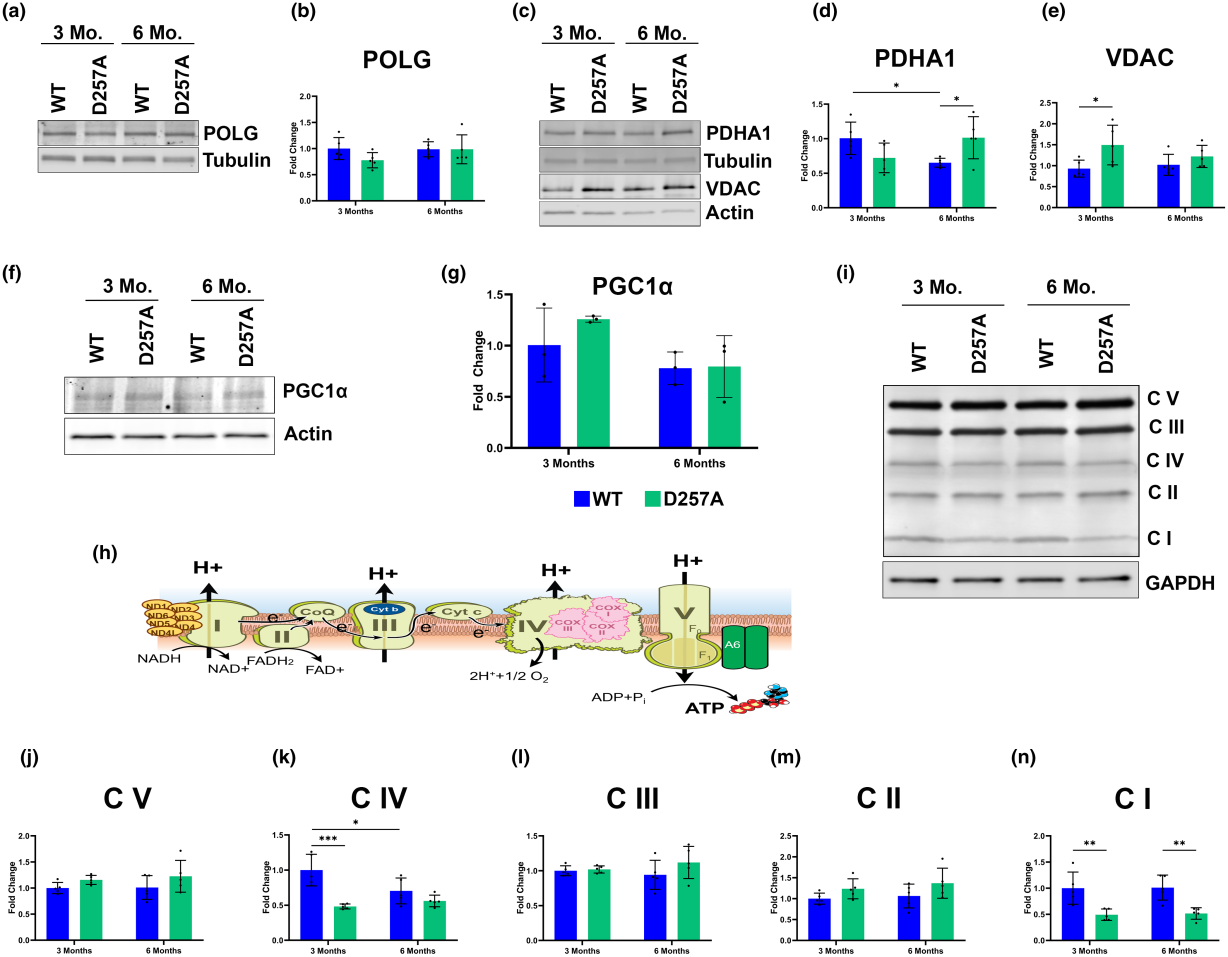
Differential expression of mt‐related proteins in the D257A mouse retina. (a) Representative immunoblot of POLG protein content in the retina. (b) Graphical representation of POLG protein content in WT and D257A mice retina. (c) Representative immunoblot of PDHA1 and VDAC. (d) Graphical representation of PDHA1 and (e) VDAC protein content. (f) Representative immunoblot of PGC1α. (g) Graphical representation of PGC1α protein content. (h) Electron transport chain indicating mt encoded subunits of complex proteins. (i) Representative immunoblot of C I‐C V. (j) Quantification of C V, (k) C IV, (l) C III, (m) C II, (n) C I normalized to GAPDH, and fold change calculated based off 3‐month WT average. Data are expressed as mean ± SD. *p ≤ 0.05, **p ≤ 0.01, ***p ≤ 0.001; two‐way ANOVA. Data points represent biological replicates; asterisks above for significance.

Several mt‐specific proteins were investigated next. Because many of the observations detected changes in the D257A occurring between 3‐ and 6‐ months of age, we proceeded to gain a deeper understanding of the biochemical changes occurring in the retina and RPE of the D257A at these ages before aging became a compounding factor. Western blot analysis of POLG protein expression was not found to be changed between ages in both mice (Figure 4a,b). Voltage‐dependent anion channel (VDAC) and pyruvate dehydrogenase (PDHA1) were also investigated. The upregulation of PDHA1 is associated with the enhancement of the mt‐mediated apoptosis pathway and the inhibition of aerobic glycolysis, promoting oxidative phosphorylation (Sun et al., 2019). PDHA1 is a mt matrix marker and was found to be significantly upregulated in 6‐month‐old D257A retina, and it was significantly decreased in WT between 3‐ and 6‐months of age (Figure 4c,d). In addition to being a commonly used mt outer membrane marker, VDAC is responsible for apoptosis by mediating the release of apoptotic factors from the mitochondria (Shoshan‐Barmatz et al., 2020). VDAC was significantly upregulated at 3 months in the D257A retina (Figure 4c,e). To determine if mt biogenesis was altered, retinas were probed for peroxisome proliferator‐activated receptor gamma coactivator 1‐alpha (PGC1α). No significant difference in PGC1α levels was observed between age in both mice (Figure 4f,g). Finally, to test the hypothesis that mtDNA mutation accumulation leads to dysfunctional components of oxidative phosphorylation complexes, NDUFB8 (Complex I, C I), SDHB (Complex II, C II), UQCRC2 (Complex III, C III), MTCO1 (Complex IV, C IV), and ATP5A (Complex V, C V) were also probed (Figure 4h,i). The levels of C V were not significantly changed in the D257A retina (Figure 4j). Complex IV levels were significantly downregulated in the D257A retina at 3 months of age compared to WT, but no significant changes were observed at 6 months. This could be because WT levels are also significantly decreased between 3‐ and 6‐months of age (Figure 4k). The levels of C III and C II were not significantly changed in D257A retinas compared to WT (Figure 4l,m). Complex I levels were significantly reduced in the D257A retinas at both ages investigated (Figure 4n).

**FIGURE 5:**
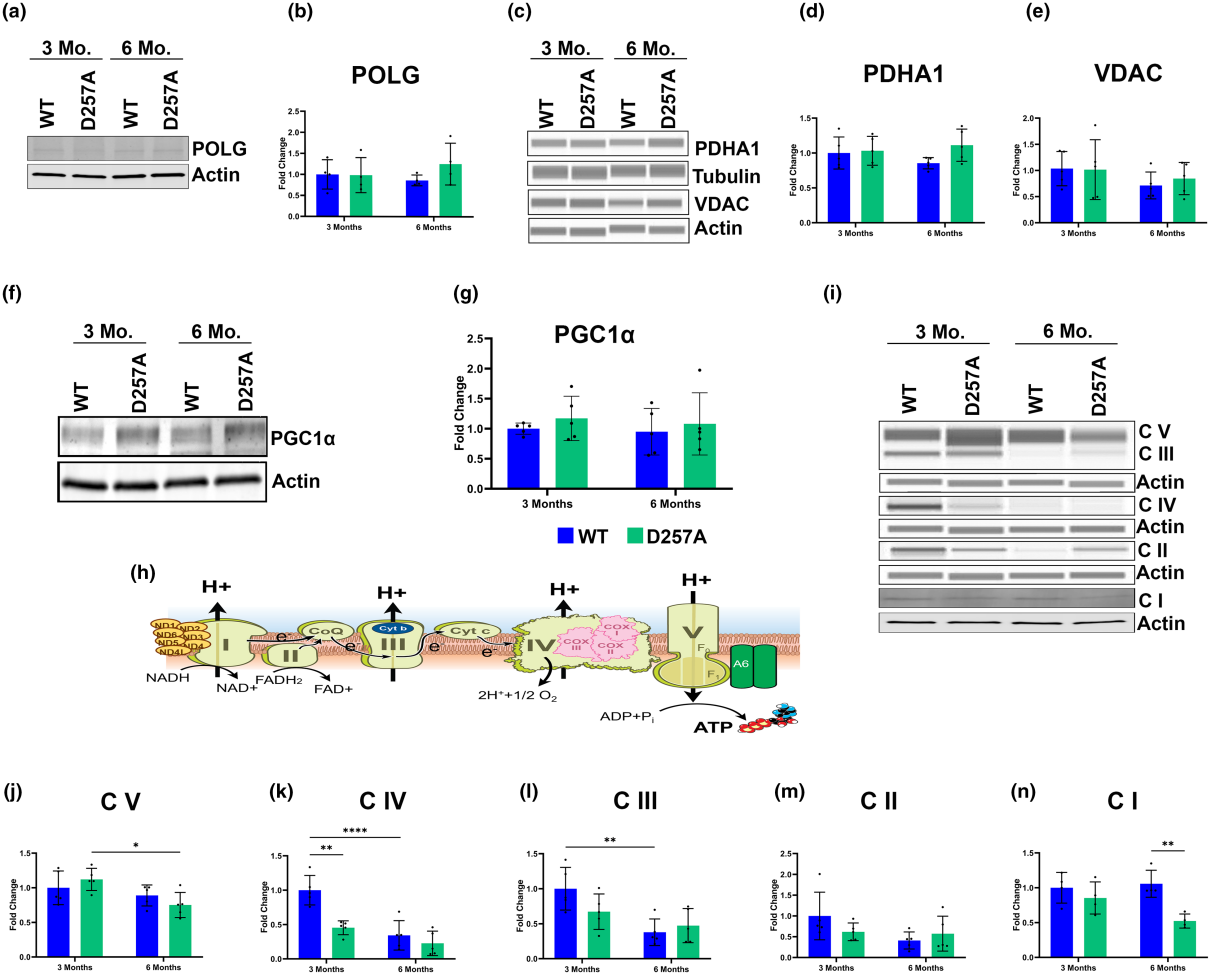
Differential expression of mt‐related proteins in the D257A mouse RPE. (a) Representative immunoblot of POLG protein content in the RPE. (b) Graphical representation of POLG protein content in WT and D257A mice RPE. (c) Representative immunoblot of PDHA1 and VDAC. (d) Graphical representation of PDHA1 and (e) VDAC protein content. (f) Representative immunoblot of PGC1α. (g) Graphical representation of PGC1α protein content. (h) Electron transport chain indicating mt encoded subunits of complex proteins. (i) Representative immunoblot of C I‐C V. (j) Quantification of C V, (k) C IV, (l) C III, (m) C II, (n) C I normalized to Actin, and fold change calculated based off 3‐month WT average. Data are expressed as mean ± SD. *p ≤ 0.05, **p ≤ 0.01, ****p ≤ 0.0001; two‐way ANOVA. Data points represent biological replicates; asterisks above for significance.

The same experiments were performed using RPE protein lysate to determine if mt health was also being compromised in the RPE. Like the observed retina phenotype, POLG protein expression in the RPE was not significantly different between ages or by comparing WT to D257A mice (Figure 5a,b). Unlike the retinal phenotype, neither PDHA1 nor VDAC increased in the D257A mice RPE (Figure 5c–e). Additionally, PGC1α levels were not significantly different between ages in both mice (Figure 5f,g). The same components of the oxidative phosphorylation complexes described above were probed in RPE lysates (Figure 5h,i). Quantifying C V levels only detected a significant decrease between D257A 3‐ and 6‐month‐old (Figure 5j). Complex IV levels were significantly reduced in the D257A RPE at 3 months compared to WT. However, WT C IV levels were significantly reduced with age (Figure 5k). Complex III displayed a decreasing trend in the 3‐month D257A RPE and a significant decrease with age when comparing 3‐ and 6‐month‐old WT (Figure 5l). C II levels were not significantly altered (Figure 5m). Finally, C I levels were significantly decreased in the D257A 6‐month RPE (Figure 5n). This data suggests that the observed changes in both retina and RPE are not due to increased or decreased POLG levels. Additionally, the increased expression of PDHA1 and VDAC at specific ages in the D257A retina could indicate apoptosis activation, explaining the significant loss in ONL thickness and cell count observed above. The significantly reduced C I and C IV levels in the retina and RPE of the D257A mouse suggest a preferential loss of these proteins over others. Since this mouse accumulates a rapid rate of mtDNA mutations, these complexes are likely preferentially disrupted because they contain the most mtDNA encoded subunits, with seven subunits in C I and three in C IV (Figures 4h and 5h, orange/pink subunits) leading to an improper assembly of the entire complex. Finally, aging plays a significant role in the expression of complex proteins, specifically in the RPE. Some complexes (C IV and C III) were significantly depleted between 3 months and 6 months in WT mice RPE, suggesting a higher susceptibility to aging‐related oxidative phosphorylation complex degeneration than the retina counterparts.

### 2.5 Oxidative stress and parkin‐mediated mitophagy do not contribute to retinal accelerated age‐related changes in the D257A mice

**FIGURE 6:**
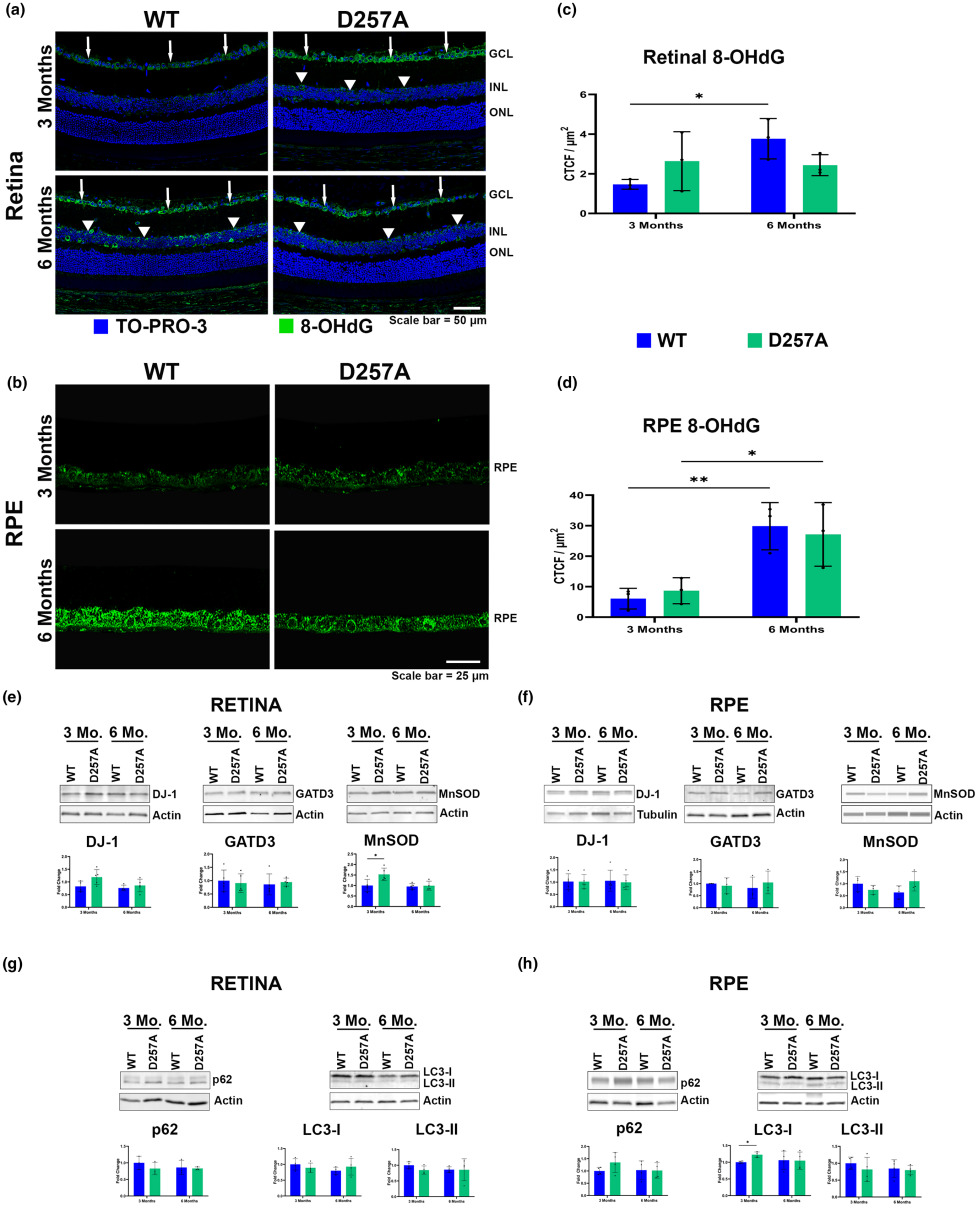
Analysis of oxidative stress, mitophagy, and autophagy in the D257A retina and RPE. (a) Representative retinal sections stained with 8‐OHdG (8‐hydroxy 2′‐deoxyguanosine). White arrows and arrowheads show elevated staining in the GCL (ganglion cell layer) and INL (inner nuclear layer), respectively. (b) Representative RPE sections stained with 8‐OHdG. (c) Graphical representation of retina 8‐OHdG staining, and (d) RPE 8‐OHdG staining. CTCF, corrected total cell fluorescence. (e) Representative retina immunoblots (f) and RPE with their respective quantifications of proteins DJ‐1, GATD3, and MnSOD. (g) Representative retina immunoblots (h) and RPE with their respective quantifications of proteins p62, LC3‐I, and LC3‐II. Data are expressed as mean ± SD. *p ≤ 0.05, **p ≤ 0.01; two‐way ANOVA. Data points represent biological replicates; asterisks above for significance.

Previous studies on D257A mice have detected tissue‐specific changes in oxidative stress‐related compounds (Hiona et al., 2010; Zsurka et al., 2018). Both retinal and RPE protein lysates of 3‐ and 6‐month‐old WT and D257A mice were tested, with no significant increase observed in protein carbonylation (Figure S3A–D). Retinal sections were probed for 4‐hydroxynonenal (4‐HNE) to determine if lipid peroxidation was impacted. No significant increase in staining was observed (Figure S3E–G), whereas the retinas of mice injected with 20 mg/kg sodium iodate showed qualitatively more staining across the entire retina (Figure S3H). Additionally, WT and D257A retinal sections were probed for 8‐hydroxy‐2′‐deoxyguanosine (8‐OHdG), a well‐established biomarker for oxidative damage to both mt and nuclear DNA (Valavanidis et al., 2009). At 3 months of age, D257A retinas exhibited a slight, but not statistically significant, increase in the labeling in the ganglion cell layer (GCL), consistent with previous literature. (Figure 6a, arrows); minor 8‐OHdG labeling is observed in the INL (Figure 6a, arrowheads). A significant increase in labeling was observed in the 6‐month‐old WT retinas compared to the 3‐month WT, with stronger staining in both the GCL and INL (Figure 6a, arrows, and arrowheads). However, this increase was not observed with aging in the D257A mice retina (Figure 6c). Moreover, 8‐OHdG did increase with age in both mice RPE (Figure 6b,d). Our data agrees with a previous study which reported that the retinal 8‐OHdG levels naturally increase with age (Wang et al., 2010), suggesting that oxidative damage is not correlated with mtDNA mutation burden but is more significantly impacted by aging. The mouse retina and RPE were probed with antibodies to proteins, including Parkinson's disease protein 7 (PARK7, DJ‐1), glutamine amidotransferase class 1 domain containing 3 (GATD3), and manganese‐dependent superoxide dismutase (MnSOD) to determine the antioxidant levels in both mice (Bonilha et al., 2015; Kowluru et al., 2006; Smith et al., 2022). A significant increase in expression was observed only in MnSOD in the 3‐month D257A retina (Figure 6e,f). Finally, parkin‐mediated mitophagy and autophagy were separately analyzed in these tissues according to previous literature (Woodall et al., 2019). The sequestosome 1 (p62) protein levels were measured to determine parkin‐mediated mitophagy, and no changes were observed (Figure 6g,h). Microtubule‐associated proteins 1A/1B light chain 3B (LC3B) were investigated in these tissues to examine autophagy activation. A significant increase in LC3‐I was observed only in 3‐month D257A RPE (Figure 6g,h). Overall, this data suggests that an increase in oxidative stress is perhaps tissue‐specific and is not correlated with mtDNA mutation burden in the retina and RPE. In addition, parkin‐mediated mitophagy and autophagy activation do not seem to be playing a role in the retina or RPE phenotypes observed.

## 3 DISCUSSION

The current study comprehensively examines the retina and RPE health of the D257A mouse model. A wide range of ages was used to delineate the contributions of normal physiological aging processes with those attributed explicitly to mtDNA mutation accumulation. We systematically demonstrate that D257A mice develop accelerated age‐related progressive retinal abnormalities. A summary of the main age‐related changes identified is shown in Table 1; additional, non‐significant findings are shown in Table S1. Our data indicate that D257A retinas display signs of morphological abnormalities and physiological dysfunction associated with mt dysfunction. Our results establish the D257A mice as a model to understand the contribution of mtDNA mutation burden in retinal degeneration.

**TABLE 1:** Summary of main age‐related morphological and functional findings in the D257A retinas.

Age	OCT	ERG	R/G opsin	Cone arrestin	RPE AF granules	RPE TEM (4800x)	RPE TEM (30kx)	Retina mt proteins	RPE mt proteins	8‐OHdG	Antioxidant proteins	Autophagy and mitophagy markers
3 Months	No observable changes in total retinal thickness	No observable changes in cell function	Non‐significant increase in staining	No observable changes in cone morphology	Significantly more granules/μm^2^	Disorganized microvilli, basal infoldings, and pigment; undigested OS and presence of vacuoles	Mt significantly shorter in length; no difference in mt number	Significant increase in VDAC; decrease in COX IV and COX I	Significant decrease in COX IV	Non‐significant increase in staining	Significantly increased MnSOD in retina	Significantly increased LC3‐I in RPE
6 Months	Thickness decreases in ONL	Decreased a‐wave, b‐wave, and light‐adapted response amplitudes	Significantly decreased staining	No observable changes in cone morphology	Non‐significant increase in granules/μm^2^ and average granule size	N/A	N/A	Significant increase in PDHA1; decrease in COX I	Significant decrease in COX I	Significantly more staining in RPE with age but not genotype	No observable changes in retina or RPE	No observable changes in retina or RPE
9 Months	Thickness decreases in ONL and total retina	Highest observed decrease in light‐adapted response amplitudes	WT loses staining, creating decreased staining difference	Significantly shorter cone cell length	Significantly larger average granule size	N/A	N/A	N/A	N/A	N/A	N/A	N/A

Our in vivo OCT analysis revealed total retinal thinning by 9 months of age, with significant thinning of the ONL of the photoreceptors observed at 6 and 9 months of age. This change could be validated by histology and attributed to the ONL thinning identified in 9‐month‐old D257A retinas.

The ERG testing detected significantly decreased a‐wave amplitude at 6 months in D257A mice, indicating a functional reduction in rod photoreceptor cells at this age. These results were corroborated by the reduced rhodopsin labeling observed in 6 and 9‐month D257A retinas. Furthermore, the cone ERG was significantly decreased at 6 and 9 months in D257A mice. These results were corroborated by a significant decrease in the red/green cone opsin labeling and a decrease in cone length, suggesting a reduction in R/G cones may underlie the photopic ERG defect.

Lipofuscin is a long‐lived intracellular inclusion body, lipid‐ and bisretinoids‐rich, and autofluorescent material that progressively accumulates in the RPE during aging and pathological conditions (Feeney, 1978). The investigation of the RPE layer of the D257A mice showed a higher abundance of AF granule accumulation in their cytoplasm. However, the TEM of D257A RPE did not detect granules with structural similarity to previously reported lipofuscin granules (Schraermeyer & Heimann, 1999). Further experiments are needed to confirm the molecular nature of these AF granules, as these are composed of something removed during tissue preparation for TEM, such as retinoids. The early changes observed in the RPE likely promote the unhealthy state of the retina at later time points. Our TEM images detected additional changes, such as basal infoldings' disorganization in the D257A RPE. The changes in these RPE structures could be driving a nutrient deficit in the RPE and, therefore, a deficit in the neural retina. Because the RPE changes were observed before other changes in the retina, we hypothesize that the RPE dysfunction may promote the retinal dysfunctions. Previous studies on D257A mice reported alopecia, anemia, reduced subcutaneous fat, kyphosis, and osteoporosis by approximately 6 months (Trifunovic et al., 2004; Vermulst et al., 2007). Thus, the RPE dysfunction observed at 3 months of age suggests a crucial role of mtDNA mutations in these cells. Moreover, determining a similar number of mitochondria in both WT and D257A RPE suggests that mt biogenesis is not altered in these cells. Our results also determined a decrease in the overall length of the mt in the D257A RPE cells. A previous study reported that the deletion of mt phosphatase phosphoglycerate mutase 5 (PGAM5), a mt Serine (Ser)/Threonine (Thr) phosphatase located in the inner mt membrane, leads to accelerated senescence by regulating mt dynamics (Yu et al., 2020). Future experiments will determine if this pathway is affected in the retina and RPE of the D257A mice.

Interestingly, oxidative stress in this model's retina and RPE cytoplasm does not appear to be elevated compared to WT but is elevated with normal physiological aging. Our data agrees with previous studies on young and aged D257A mice, which did not detect a significant increase in levels of oxidative stress markers (Hiona et al., 2010; Kujoth et al., 2005; Mott et al., 2001; Trifunovic et al., 2005). Probing of MnSOD displayed significantly increased expression in lysates of the D257A retina, suggesting that there may be a significant increase in mt‐related oxidative stress. Mitophagy is the process by which dysfunctional mt are expelled and replaced with normal functioning mt. This process has already been thoroughly characterized as playing a role in RPE heterogeneity (Datta et al., 2023). Mitophagy can be mediated by the PINK1/Parkin‐mediated and receptor‐mediated mitophagy pathways (D'Arcy, 2024). Unlike previous studies reporting that parkin‐mediated mitophagy was altered in POLG hearts (Woodall et al., 2019), our data suggests that this mitophagy pathway is unaltered in the retinas of the D257A mice. Further experiments will be needed to determine if the receptor‐mediated mitophagy pathway is altered in the D257A retinas.

Finally, analysis of mt markers, morphology, and ETC complex proteins revealed substantial differences. The changes in PDHA1 and VDAC levels in the retina indicate the activation of the apoptotic pathway, signifying a possible method for the observed ONL thinning. In the all‐cone Nrl KO mouse, PDHA1 was found to be upregulated due to the high reliance of cone photoreceptor cells on pyruvate metabolism (Li et al., 2020), a phenomenon that could be similarly present as a compensatory mechanism in D257A cone cells. Additionally, VDAC, a protein that is associated with the mt permeability transition pore and highly localized to photoreceptor and ganglion cells, could be involved in mt swelling or other processes as a result of ATP depletion or calcium overload (Gincel et al., 2002). The decline in cristae density and normal mt shape in the RPE could also indicate declining mt function and energy production. Our data agrees with previously reported data from the D257A mouse, supporting the hypothesis that mtDNA mutations can promote tissue dysfunction through the loss of critical irreplaceable cells due to the activation of apoptosis (Kujoth et al., 2007). The unaltered levels of PGC1α, known to regulate cellular antioxidant homeostasis and mt biogenesis together with our quantification of mt by TEM suggests that D257A retinas do not have increased levels of mt turnover at baseline conditions.

Our western blot analysis revealed a preferential decline in ETC proteins between D257A and WT. Specifically, C I and C IV were most impacted in the retina and RPE of 3‐month D257A mice, while C I was most impacted in the 3‐ and 6‐month‐old retinas and 6‐month‐old RPE. Interestingly, these complexes contain the most mtDNA‐encoded subunits, with seven and three proteins encoded by mtDNA, respectively these data corroborate previously published data indicating that the primary impact of dysfunctional POLG encompasses a specific reduction in C I and C IV (Dai et al., 2013; Hiona et al., 2010). It is important to note that the strong correlation between loss of complex protein expression and aging could be due to the background spontaneous mutation in nicotinamide nucleotide transhydrogenase (NNT) found in C57Bl/6J mice. This mutation was previously associated with loss and gain of expression that seems tissue‐specific for many of the mt redox proteins (Ronchi et al., 2013; Williams et al., 2021). Since the D257A model has been developed in the C57Bl/6J background, this mechanism must not be ruled out to influence the mt complex proteins analyzed.

Although this study provides a comprehensive baseline characterization of many different retinal and RPE phenotypes in this mouse model, it did not identify a specific functional mechanism behind the observed decline in retinal health. The most likely origin of the decaying retina is the implication of mt dysfunction, which is apparent in this mouse model. Since our findings do not support overall increased cytoplasmic oxidative stress and impaired parkin‐mediated mitophagy in the ages analyzed, the observed defects are possibly a consequence of altered receptor‐mediated mitophagy pathway and proteasomal activity, nutrient deprivation, or acute immune responses, which were not investigated. The disruption of mt observed at an early age in the RPE of this model could indicate a respiratory chain inhibition and, therefore, a nutrient deprivation in the neural retina that supports the idea of a “metabolic switch” where RPE cells start consuming metabolites initially intended to be supplied to the retina (Hurley, 2021). Damage to mtDNA in this model may lead to an acute immune response in the retinal tissue. The cGAS/STING pathway is a well‐studied cytosolic DNA sensor system that has been identified to recognize mtDNA and initiate an acute immune response in these cells, leading to inflammatory‐induced damage that can be therapeutically targeted (Kim et al., 2023). Future studies of this model should include an investigation into all these well‐studied mechanisms of retinal degeneration to determine the impact of mtDNA mutations in these pathways.

The observation of a relatively mild retinal degeneration phenotype in aged D257A mice described here could be related to increased (parkin‐independent) mt turnover, proteasomal activity in D257A retinas, senescence, or a high tolerance (threshold) for mtDNA mutations, and mt deficiencies in the retina. A previous analysis of Tfam‐deficient retinas suggested that the RPE cells are largely resistant to cell death, autophagy, and senescence under conditions of severe OXPHOS deficiency (Zhao et al., 2011). Moreover, previously described mice with decreased mt function display similar mild retinal pathology (Gurubaran et al., 2024; Xu et al., 2020; Yu et al., 2020).

## 4 METHODS

### 4.1 Mice

All procedures were approved by the Institutional Animal Care and Use Committee (ARC 2021‐2191) of the Cleveland Clinic. Twelve‐week‐old D257A and WT mice were purchased from the Jackson Laboratory. Experiments were conducted on male and female littermate Polg^WT/WT^ (wild‐type, WT), Polg^WT/D257A^ (heterozygous), and D257A^/D257A^ (D257A). Mice were genotyped as previously described (Kujoth et al., 2005). To not introduce mtDNA mutation burden during birth and development, male heterozygous D257A mice were crossed with C57Bl/6J females to generate wild‐type (WT), heterozygous D257A^/WT^ and homozygous D257A^/D257A^ experimental mice. Mice were housed in individually ventilated cages in a 14‐h light/10‐h dark cycle and were provided regular chow and water ad libitum. Mice were tested, and tissue collected at ~3 hours after light input to avoid circadian variations.

### 4.2 In vivo imaging of retinas

Imaging by spectral‐domain optical coherence tomography (SD‐OCT) (Leica Envisu R2210 UHR, Leica Microsystems) was carried out following sodium pentobarbital anesthesia and pupil dilation with 10% phenylephrine HCL and 1% tropicamide and topical anesthesia 0.5% proparacaine HCl as previously described (Singh et al., 2020). Volumetric rectangular scans (1.8 mm × 1.8 mm, 500 a‐scans × 500 b‐scan × 3 frames) were captured using 55° mouse OCT lens. Thickness measurements of each eye were performed using automated mouse retina segmentation software (Diver 1.4, Bioptigen Inc., InVivoVue Imaging Software). The scan parameter used for heatmaps were 1.8 mm diameter scan, 500 b‐scans, 500 a‐scans/b‐scans, 3 frames average. The volume intensity projection (VIP) is segmented into eight layers, and InVivoVue measures layer thickness at 9 × 9 locations (excluding the 81st at the ONH center). Software averages points within an ETDRS grid sector, generating a heat map report with an averaged heat map and segmented VIP layers. The grid, with radii at 100, 300, and 600 μm, calculates the average thickness for sector‐contained points. Innermost sector results (100 μm radius) are omitted due to ONH‐centered scans.

### 4.3 In vivo testing of retinal function

Retinal functions of mice were assayed after overnight dark adaptation as previously described (Aiello et al., 2023). Mice were anesthetized with 65 mg/kg sodium pentobarbital. Eye drops were used to anesthetize the cornea (1% proparacaine HCl) and to dilate the pupil (2.5% phenylephrine HCl, 1% tropicamide, and 1% cyclopentolate HCl). Mice were placed on a temperature‐regulated heating pad throughout the recording session. In brief, ERGs were recorded in response to strobe flash stimuli presented in the dark by an Espion E3 ColorDome Full Field Ganzfeld (Diagnosys). An Ag/AgCl electrode in contact with the cornea was referenced to a needle electrode placed in the mouth of the mouse, and a ground lead was placed in the tail. Scotopic responses were obtained in the dark with 10 steps of a blue‐green flash stimulus, ranging from −3.6 log cd. s/m^2^ to 2.1 log cd. s/m^2^. The duration of the inter‐stimulus intervals increased from 4 s for low‐luminance flashes to 90 s for the highest stimuli. Two minutes following the scotopic ERG, a 10‐s blue‐green stimulus (5 cd/m^2^) was presented to elicit the c‐wave. After 7 min of light adaptation, cone ERGs were recorded with strobe‐flash stimuli (−1 to 2 log cd·s/m^2^) superimposed on the adapting field. Amplitude of the a‐wave was measured at 8.3 ms following the stimulus. The b‐wave amplitude was calculated by summing the amplitude of the a‐wave at 8.3 ms with the peak of the waveform after the oscillatory potentials (≥40 ms). Light‐adapted response amplitudes were calculated by summing the peak of the waveform with the amplitude at 8.3 ms.

### 4.4 Histology and transmission electron microscopy (TEM) of retinas

Enucleated eyes were fixed overnight at 4°C by immersion in 2% paraformaldehyde, 2.5% glutaraldehyde, and 5% CaCl_2_ in 0.1 M cacodylate buffer. After removing the anterior segments under a dissecting microscope, eyecups were processed for epon embedding as previously described (Bonilha et al., 2015). For bright‐field microscopy, semi‐thin sections were cut using a diamond histotech knife (DiATOME), collected on glass slides, and stained with toluidine blue. Sections were imaged with a THUNDER 3D Assay inverted microscope equipped with a Leica K3C Color camera (Leica Microsystems). High‐magnification images were acquired within 200 μm of the optic nerve head (on both sides). Images were exported to ImageJ software (National Institute of Health) and calibrated using an embedded reference scale. The retinal layer thickness was delineated as delineated using the freehand line and measured in triplicate to obtain a mean thickness for each mouse. Three measurements were performed throughout each acquired image within 2 sections/eye. For TEM, the same block of epon‐embedded samples ultra‐thin sections of 85 nm were cut with a diamond knife, stained with uranyl acetate and lead citrate, and observed with a Tecnai G2 SpiritBT, electron microscope operated at 60 kV.

### 4.5 Retinal immunohistochemistry

Enucleated eyes were fixed overnight at 4°C by immersion in 4% paraformaldehyde in D‐PBS and sequentially infused with sucrose and Tissue‐Tek O.C.T Compound (Sakura Finetek). Cryosections (8 μm) were cut on a cryostat HM 505E (Microm) equipped with a CryoJane Tape‐Transfer system (Leica Inc.). For labeling, sections were washed with PBS, blocked in PBS supplemented with 1% BSA (PBS/BSA) and 0.1% Triton‐X100 for 30 min, and incubated with primary followed by secondary antibodies coupled to Alexa fluorophores. Nuclei were labeled with TO‐PRO‐3 (Thermo Fisher Scientific Inc). Sections were imaged using a laser scanning confocal microscope (Leica TCS‐SP8) using the same acquisition parameters for each channel in the Leica confocal software LAS‐X. Primary antibodies used can be found in the supplemental information (Table S2). Autofluorescence of unlabeled cryosections was performed and analyzed using confocal microscopy in the green channel (FITC filter: 490 nm excitation/519 nm emission) and red channel (TRITC filter: 550 nm excitation/570 nm emission). Autofluorescence was overlaid on differential interference contrast (DIC) images. Signal intensity was quantified within 200 μm of the optic nerve head, and the corrected total cell fluorescence (CTCF) was calculated for each area according to the following formula: CTCF = integrated density – (area of selected cell x mean fluorescence of background readings).

### 4.6 Protein extraction and western blotting

Mechanically isolated retinas and RPE were lysed in RIPA buffer (Alfa Aesar) containing a protease and phosphatase inhibitor cocktail (Sigma‐Aldrich). Retinas were sonicated twice for 15 s while RPE was passed through a 27 1/2 G syringe needle, incubated on ice, and vortexed every 5 min for 20 min. Both lysates were centrifuged for 10 min at 14000 rpm at 4°C, after which the supernatants were collected for western blotting. Protein quantification was performed using the MicroBCA Kit (Thermo Fisher Scientific Inc), followed by retinal protein (30 μg) separation via 4–20% Novex Tricine SDS‐ PAGE (ThermoFisher Scientific Inc) and transfer to PVDF membranes (Immobilon‐ FL; Merck Millipore). Primary antibodies used can be found in the supplemental information (Table S2), followed by washing and incubation with anti‐mouse IRDye®680RD, anti‐rabbit IRDye®680RD, and anti‐mouse IRDye®800CW (all from LI‐COR Biosciences). Immunoreactive signals were visualized using Oddessey CLx (LI‐COR Biosciences). The protein levels were quantified using ImageJ software. Housekeeping proteins were used as internal control, and 3‐month‐old WT mice averages were used to calculate fold changes. For RPE samples that do not contain high protein content, Jess Simple Western was used to conserve protein lysate. Jess, an automated western nano‐assay system (ProteinSimple, Bio‐techne) was used with internal standards to quantify the expression of different proteins in RPE lysates. The lysates were diluted with 0.1x sample buffer and mixed with fluorescent standards and 400 mM of dithiothreitol to reach a final concentration of 0.9 μg/μL. 2.7 μg of total protein was loaded from each sample onto the Jess assay plate. The 12–230 KDa fluorescence separation module was used in this study with the 25‐well capillary system. Antibody diluent, anti‐mouse NIR and anti‐rabbit NIR from ProteinSimple were used to set up the assay plate, as recommended by the instrument manual. The samples were separated at 375 V for 25 min and subjected to 30 min of blocking, 90 min of primary antibody incubation and 60 min of secondary antibody incubation. Compass for simple western v.6.3 software was used to analyze the data.

### 4.7 Protein carbonylation detection

Mechanically isolated retinas and RPE were lysed in RIPA buffer as described above. Protein carbonyl residues in were quantified in 10 μg of the cell lysates using the Protein Carbonyl Assay Kit (Abcam). Derivatization and neutralization were carried out according to the manufacturer's protocol. A parallel set of lysates was treated with the 1x Derivatization Control solution. Individual oxidized proteins were resolved by SDS‐PAGE on 4%–20% polyacrylamide gel gradient gel (BioRad). Resolved proteins were transferred to PVDF membranes at 75 V for 2 h. The membranes were blocked using 5% skimmed milk in PBS for 2 h and incubated with primary anti‐DNP rabbit antibody (1:5000) overnight at 4°C, followed by incubation with anti‐rabbit IRDye® 800CW. The same membranes were next incubated with REVERT total protein stain (LI‐COR). Signal intensity in the derivatized lanes and the control lanes was normalized with total protein signal in the respective lanes.

### 4.8 Statistical analysis

Data were analyzed using GraphPad Prism v10.1.1 (GraphPad Software) and are presented as the mean ± standard deviation (SD). Two‐way ANOVA with Tukey's multiple comparisons test (main row effect) and (simple effects within columns) and unpaired, two‐tailed Student's *t*‐test were used to determine statistical significance between groups with an alpha value of 0.05. *p* ≤ 0.05 was considered statistically significant.

## AUTHOR CONTRIBUTIONS

J.S. and V.L.B. conceptualized and designed the study. R.S. and Q.R.C. performed and helped analyzing the in vivo imaging while I.S.S. performed the ERGs. T.M.S. performed and helped analyzing the Jess Western. A.M. and P.F. helped preparing figures and collecting tissue, respectively. J.S. wrote the manuscript, and all authors contributed to the editing and finalization of the manuscript.

## FUNDING INFORMATION

This work was supported by the National Institutes of Health [grant number P30EY025585]; a challenge grant from the Research to Prevent Blindness; a Cleveland Eye Bank Foundation Grant awarded to the Cole Eye Institute, and Cleveland Clinic Foundation startup funds, and the Timken Foundation. A training grant provided by the National Eye Institute [T32EY024236] and a fellowship [F31EY035133]. Department of Veteran's Affairs (BX005844).

## CONFLICT OF INTEREST STATEMENT

The authors have no conflict of interest to declare.

## Supporting information


Table S1.

Table S2.

**Figure S1:** In vivo morphological and functional retinal characterization of D257A retinas. (A) Graphical representation of retinal cell layer thicknesses. INL, inner nuclear layer; IPL, inner plexiform layer; IS, inner segments; OPL, outer plexiform layer; OS, outer segments; RNFL, retina nerve fiber layer; RPE, retinal pigment epithelium. (B) Graphical representation of ERG b‐wave to a‐wave ratio. Data are expressed as mean ± SD. *p ≤ 0.05, **p ≤ 0.01; two‐way ANOVA; Data points represent biological replicates; asterisks above for significance.

**Figure S2:** Loss of normal retinal morphology and essential proteins in the D257A mouse. (A) Immunofluorescence staining of rod photoreceptor outer segment marker rhodopsin and bipolar cell synapse marker PKCα. (B) Graphical representation of rhodopsin and PKCα staining. (C) Representative electron micrographs of 3‐month WT and D257A photoreceptors. CC, connecting ciliumCIS, cone inner segment; M, mitochondria; OS, outer segment; RIS, rod inner segment. (D) Representative immunoblot and the respective quantification of PDE6C. Data are expressed as mean ± SD. * *p* ≤ 0.05; two‐way ANOVA; Data points represent biological replicates; asterisks above for significance.

**Figure S3:** Analysis of protein carbonylation and lipid peroxidation in the D257A retina and RPE. (A) Protein carbonylation assay of WT and D257A total retina (B) and RPE protein lysate. (C) Graphical representation of observed retinal DNPH (2, 4‐Dinitrophenylhydrazine) (D) and RPE normalized to total protein content. (E) Immunofluorescence staining of 4‐HNE. Arrowheads pointing to abundant staining in GCL, OPL, IS, and RPE. (F) Graphical representation of retina (G) and RPE 4‐HNE staining quantification. (H) Positive control section of sodium iodate‐injected C57 mouse retina stained with 4‐HNE. Arrowheads point to increased areas of 4‐HNE‐positive cells. Data are expressed as mean ± SD; two‐way ANOVA; Data points represent biological replicates.

## Data Availability

Any information required to reanalyze the data reported in this paper is available from the lead contact upon request.
