# Supplementary material for: Modeling aging and retinal degeneration with mitochondrial DNA mutation burden
Source: Aging Cell. 2024 Aug 29;23(11):e14282. doi: 10.1111/acel.14282 (PMC11561647; doi:10.1111/acel.14282)
Supplement: Supplementary file 1 — Table S1. [file ACEL-23-e14282-s001.docx]

| **Age** | **OCT** | **ERG** | **PKCα** | **Rhodopsin** | **Retina TEM** | **PDE6C** | **DNPH** | **4-HNE** |
| --- | --- | --- | --- | --- | --- | --- | --- | --- |
| **3 Months** | No observable changes in retinal cell thickness | b-wave to a-wave ratio not significant | No observable changes in staining | Non-significant increase in staining | Cones and Rods mitochondria disorganized | No decrease in protein levels | No observable changes in protein carbonylation | No observable changes in lipid peroxidation |
| **6 Months** | Significantly decreased RPE thickness | b-wave to a-wave ratio not significant | No observable changes in staining | Non-significant decrease in staining | N/A | Non-significant decrease in protein levels | No observable changes in protein carbonylation | No observable changes in lipid peroxidation |
| **9 Months** | Significantly decreased RNFL and OS thickness | b-wave to a-wave ratio not significant | Significantly decreased staining compared to 3 month-old mutant | Significantly decreased staining compared to 3 month-old mutant | N/A | N/A | N/A | N/A |

**TABLE S1: Summary of additional age-related morphological and functional findings in the D257A retinas.**
