# Supplementary material for: Modeling aging and retinal degeneration with mitochondrial DNA mutation burden
Source: Aging Cell. 2024 Aug 29;23(11):e14282. doi: 10.1111/acel.14282 (PMC11561647; doi:10.1111/acel.14282)
Supplement: Supplementary file 2 — Table S2. [file ACEL-23-e14282-s004.docx]

**TABLE S2: List of antibodies, dilutions and applications used in this study.**

| **Antibody** | **Company** | **Catalog #** | **Host** | **Dilution** | **Use** |
| --- | --- | --- | --- | --- | --- |
| α-tubulin | Cell Signaling | 2144S | Rabbit | 1:5000 | WB |
| β-actin | Cell Signaling | 8H10D10 | Mouse | 1:5000 | WB |
| β-actin | Cell Signaling | 4970S | Rabbit | 1:5000 | WB |
| Cone Arrestin | Sigma | AB15282 | Rabbit | 1:250 | IHC |
| DJ-1 | Novus | NB300-270 | Rabbit | 1:1000 | WB |
| DNA Polymerase gamma | AbClonal | 21314 | Rabbit | 1:500 | WB |
| GAPDH | Proteintech | 10494-1-AP | Rabbit | 1:5000 | WB |
| GATD3 | Origene | TA338921 | Rabbit | 1:1000 | WB |
| LC3B I & II | Cell Signaling | 2775S | Rabbit | 1:500 | WB |
| MnSOD | Abcam | Ab13533 | Rabbit | 1:1000 | WB |
| PDE6C | Abcepta | AP9728c | Rabbit | 1:500 | WB |
| PDHA1 | Thermo Fisher | 459400 | Mouse | 1:1000 | WB |
| PGC1α | Novus | NBP1-04676 | Rabbit | 1:500 | WB |
| PKCα | Santa Cruz | SC-8393 | Mouse | 1:200 | IHC |
| P62 | Abcam | Ab56416 | Mouse | 1:750 | WB |
| Red / Green Opsin | Sigma | AB5405 | Rabbit | 1:500 | IHC |
| Rhodopsin | Abcam | Ab5417 | Mouse | 1:1000 | IHC |
| Total OXPHOS | Abcam | Ab110413 | Rodent | 1:250 | WB |
| VDAC | Thermo Fisher | PA1-954A | Rabbit | 1:1000 | WB |
| 4-HNE | Thermo Fisher | MA5-27570 | Mouse | 1:500 | IHC |
| 8-OHdG | Santa Cruz | SC-66036 | Mouse | 1:100 | IHC |

| **Antibody** | **Company** | **Catalog #** | **Host** | **Dilution** | **Use** |
| --- | --- | --- | --- | --- | --- |
| β-actin | Santa Cruz | SC-47778 | Mouse | 1:10 | Jess |
| MnSOD | Abcam | Ab13533 | Rabbit | 1:5 | Jess |
| PDHA1 | Thermo Fisher | 459400 | Mouse | 1:10 | Jess |
| Total OXPHOS | Abcam | Ab110413 | Rodent | 1:5 | Jess |
| VDAC | Thermo Fisher | PA1-954A | Rabbit | 1:25 | Jess |
