# Supplementary figures and images for: Modeling aging and retinal degeneration with mitochondrial DNA mutation burden
Source: Aging Cell. 2024 Aug 29;23(11):e14282. doi: 10.1111/acel.14282 (PMC11561647; doi:10.1111/acel.14282)

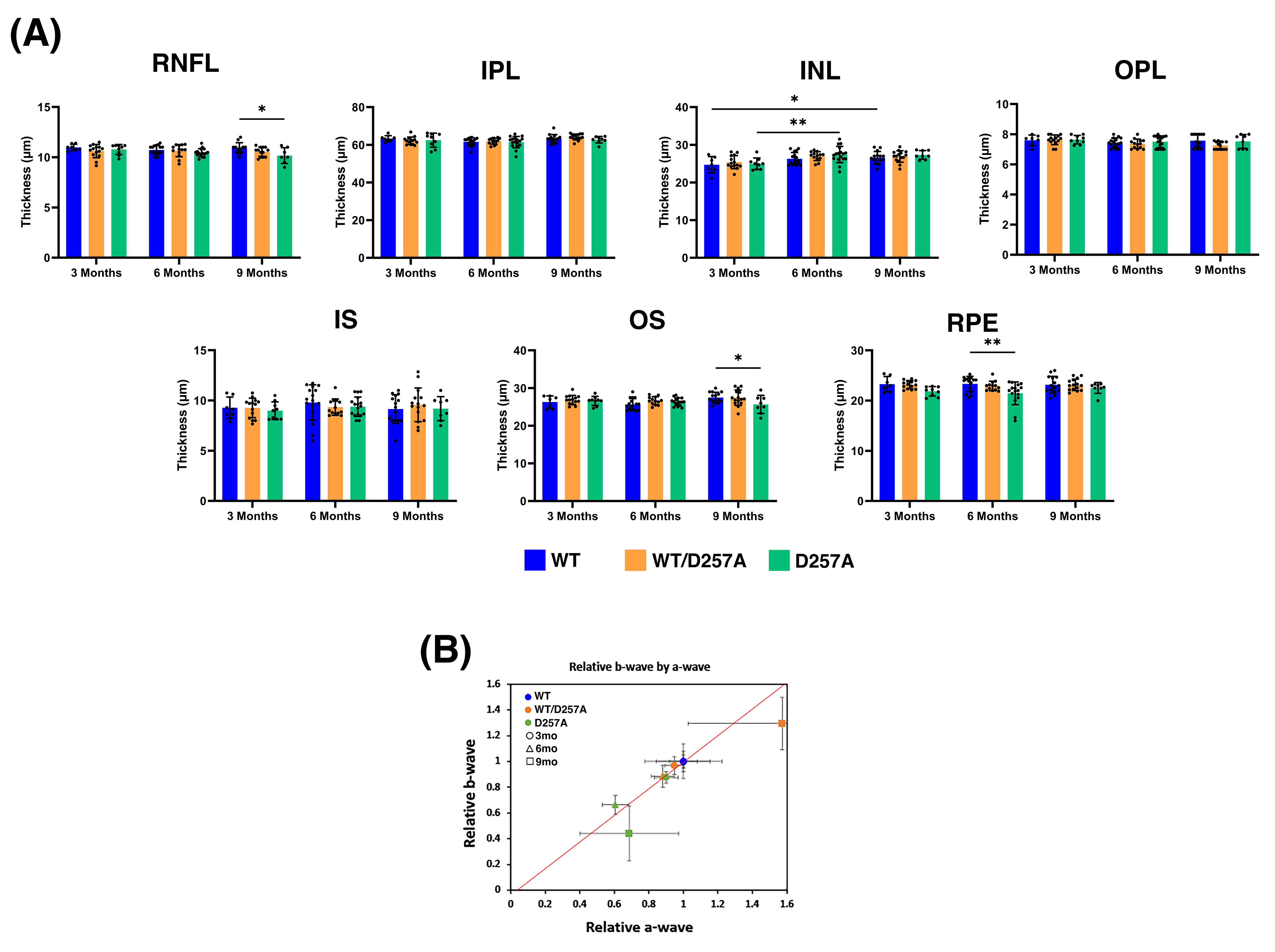

Supplement: Supplementary file 3 — Figure S1: In vivo morphological and functional retinal characterization of D257A retinas. (A) Graphical representation of retinal cell layer thicknesses. INL, inner nuclear layer; IPL, inner plexiform layer; IS, inner segments; OPL, outer plexiform layer; OS, outer segments; RNFL, retina nerve fiber layer; RPE, retinal pigment epithelium. (B) Graphical representation of ERG b‐wave to a‐wave ratio. Data are expressed as mean ± SD. *p ≤ 0.05, **p ≤ 0.01; two‐way ANOVA; Data points represent biological replicates; asterisks above for significance. [file ACEL-23-e14282-s005.tif]

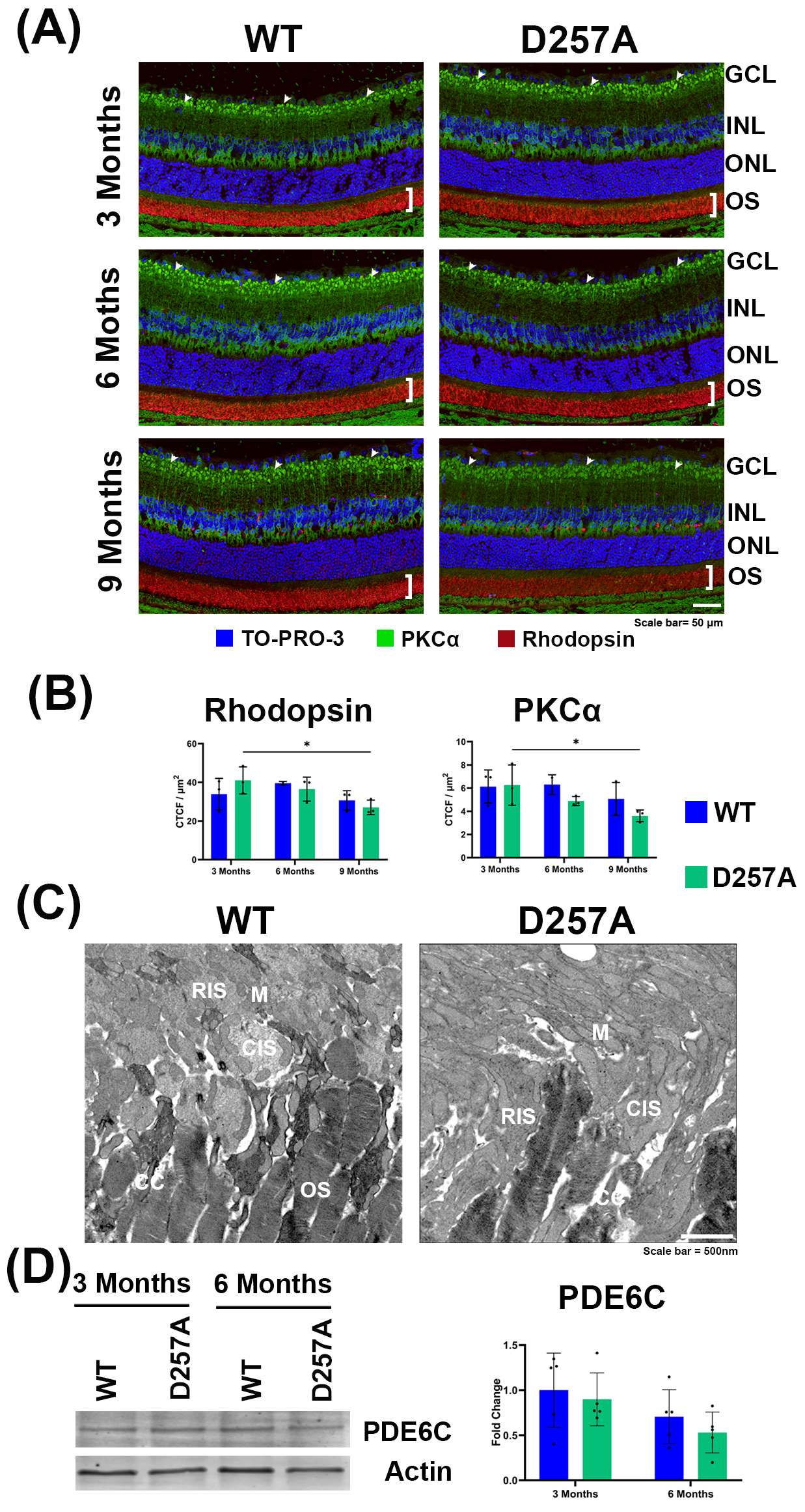

Supplement: Supplementary file 4 — Figure S2: Loss of normal retinal morphology and essential proteins in the D257A mouse. (A) Immunofluorescence staining of rod photoreceptor outer segment marker rhodopsin and bipolar cell synapse marker PKCα. (B) Graphical representation of rhodopsin and PKCα staining. (C) Representative electron micrographs of 3‐month WT and D257A photoreceptors. CC, connecting ciliumCIS, cone inner segment; M, mitochondria; OS, outer segment; RIS, rod inner segment. (D) Representative immunoblot and the respective quantification of PDE6C. Data are expressed as mean ± SD. * p ≤ 0.05; two‐way ANOVA; Data points represent biological replicates; asterisks above for significance. [file ACEL-23-e14282-s003.tif]

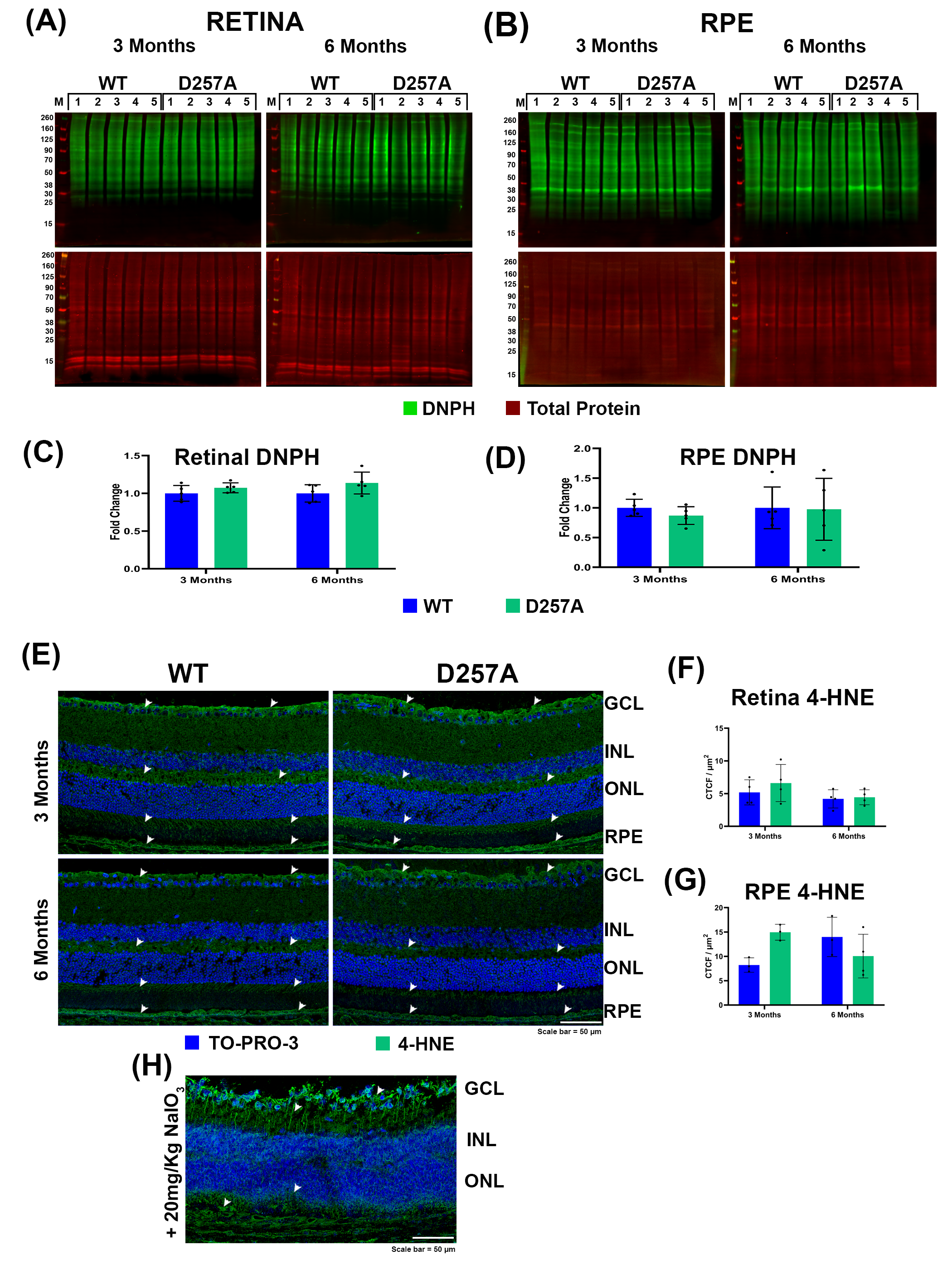

Supplement: Supplementary file 5 — Figure S3: Analysis of protein carbonylation and lipid peroxidation in the D257A retina and RPE. (A) Protein carbonylation assay of WT and D257A total retina (B) and RPE protein lysate. (C) Graphical representation of observed retinal DNPH (2, 4‐Dinitrophenylhydrazine) (D) and RPE normalized to total protein content. (E) Immunofluorescence staining of 4‐HNE. Arrowheads pointing to abundant staining in GCL, OPL, IS, and RPE. (F) Graphical representation of retina (G) and RPE 4‐HNE staining quantification. (H) Positive control section of sodium iodate‐injected C57 mouse retina stained with 4‐HNE. Arrowheads point to increased areas of 4‐HNE‐positive cells. Data are expressed as mean ± SD; two‐way ANOVA; Data points represent biological replicates. [file ACEL-23-e14282-s002.tif]
